# Anti-RGS8 paraneoplastic cerebellar ataxia is preferentially associated with a particular subtype of Hodgkin’s lymphoma

**DOI:** 10.1007/s00415-024-12618-4

**Published:** 2024-08-29

**Authors:** Elise Peter, Nicolas Lundahl Ciano-Petersen, Le-Duy Do, Jimmy Perrot, Thomas Ngo, John Pluvinage, Christopher M. Bartley, Kelsey C. Zorn, Ramona Miske, Madeleine Scharf, Macarena Villagrán-García, Antonio Farina, Véronique Rogemond, Jean-Christophe Antoine, Christine Tranchant, Valérie Dubois, Joseph L. DeRisi, Samuel J. Pleasure, Michael R. Wilson, Jeffrey M. Gelfand, Alexandra Traverse-Glehen, Jérôme Honnorat, Virginie Desestret

**Affiliations:** 1 French Reference Center on Paraneoplastic Neurological Syndromes and Autoimmune Encephalitis, Hospices Civils de Lyon, Hôpital Neurologique, Bron, France; 2 MeLiS – UCBL – CNRS UMR 5284 – INSERM U1314, Université de Lyon, Université Claude Bernard Lyon 1, Lyon, France; 3 Biomedical Research Institute of Málaga (IBIMA) and Platform of Nanomedicine (BIONAND), Málaga, Spain; 4 Hospices Civils de Lyon, Service d’Anatomie Pathologique, Centre Hospitalier Lyon Sud, Lyon, France; 5 Weill Institute for Neurosciences, Department of Neurology, University of California, San Francisco, San Francisco, CA USA; 6 Weill Institute for Neurosciences, Department of Psychiatry and Behavioral Sciences, University of California, San Francisco, San Francisco, CA USA; 7 Department of Biochemistry and Biophysics, University of California, San Francisco, San Francisco, CA USA; 8 Unit for Experimental Immunology, Affiliated to EUROIMMUN Medizinische Labordiagnostika AG, Luebeck, Germany; 9 Université Jean Monnet, Saint-Etienne, France; 10 Département de Neurologie, Centre Hospitalier Universitaire de Saint Etienne, Saint-Etienne, France; 11 Service de Neurologie, Hôpital de Hautepierre, Strasbourg, France; 12 Faculté de Médecine, Fédération de Médecine Translationnelle, Strasbourg, France; 13 Institut de Génétique et de Biologie Moléculaire et Cellulaire (IGBMC), INSERM-U964/CNRS-UMR7104/Université de Strasbourg, Illkirch, France; 14 Laboratoire d’étude du HLA, Etablissement Français du Sang Auvergne-Rhône-Alpes, Lyon, France; 15 Chan Zuckerberg Biohub SF, San Francisco, CA USA

**Keywords:** Anti-RGS8 antibodies, Cerebellar ataxia, Paraneoplastic neurological syndrome, B-cell lymphoma, Nodular lymphocyte-predominant Hodgkin lymphoma (NLPHL), Onconeural antigen

## Abstract

Ataxia with anti-regulator of G-protein signaling 8 autoantibodies (RGS8-Abs) is an autoimmune disease recently described in four patients. The present study aimed to identify other patients with RGS8-Abs, describe their clinical features, including the link between RGS8-related autoimmune cerebellar ataxia (ACA) and cancer. Patients with RGS8-Abs were identified retrospectively in the biological collections of the French Reference Center for Paraneoplastic Neurological Syndrome and the University of California San Francisco Center for Encephalitis and Meningitis. Clinical data were collected, and cerebrospinal fluid, serum, and tumor pathological samples were retrieved to characterize the autoantibodies and the associated malignancies. Only three patients with RGS8-Abs were identified. All of them presented with a pure cerebellar ataxia of mild to severe course, unresponsive to current immunotherapy regimens for ACA. Two patients presented with a Hodgkin lymphoma of the rare specific subtype called nodular lymphocyte-predominant Hodgkin lymphoma, with very mild extension. Autoantibodies detected in all patients enriched the same epitope on the RGS8 protein, which is an intracellular protein physiologically expressed in Purkinje cells but also ectopically expressed specifically in lymphoma cells of patients with RGS8-related ACA. The present results and those of the four cases previously described suggest that RGS8-Abs define a new paraneoplastic neurological syndrome of extreme rarity found mostly in middle-aged males that associates pure cerebellar ataxia and a particular lymphoma specifically expressing the RGS8 antigen. As in other paraneoplastic ACA with intracellular antigen, the disease course is severe, and patients tend to exhibit a poor response to immune therapy.

**Supplementary Information:**

The online version contains supplementary material available at 10.1007/s00415-024-12618-4.

## Introduction

Antibodies against regulator of G-protein signaling 8 (RGS8-Abs) have recently been described in four patients with ACA [1]. Among them, two patients presented a concomitant B-cell lymphoma (one with Hodgkin’s Lymphoma (HL) and one with B-cell lymphoma of the stomach), and no data regarding tumor association was available in the two other patients; it remains to be defined whether ACA with RGS8-Abs is a paraneoplastic syndrome [1]. An additional anti-RGS8 case was reported in a study on the utility of protein microarrays for autoantibodies detection [2]. Lymphoma was suspected in this patient but not proved. The aim of the present study was to identify other patients with RGS8-Abs to describe their clinical features as well as the immunological features of RGS8-Abs.

## Material and methods

### Identification of patients with RGS8-Abs

Patients with RGS8-Abs were identified retrospectively in the biological collection of the French Reference Center on Autoimmune Encephalitis (AE) and Paraneoplastic Neurological Syndrome (PNS) (n = 27,191 samples) and that of the University of California San Francisco (UCSF) using a two-step analysis. An indirect immunofluorescence (IF) on rat brain sections was performed as a screening technique, as previously described [3]. To confirm the positive result, a cell-based assay (CBA) was conducted as previously described [4] with patients’ serum (1:100) or CSF (1:10), or a commercial rabbit monoclonal anti-Myc antibody. References of the antibodies are provided in the supplementary methods. Samples were considered positive when both techniques (IF and CBA) gave a positive result. In addition, RGS8-Abs were detected using a line blot developed by EUROIMMUN AG (Lübeck, Germany). To assess RGS8-Ab specificity, the serum and CSF of a validation cohort that included 128 autoimmune encephalitis, 83 autoimmune cerebellar ataxia, and 165 patients with neurodegenerative disorders were tested.

### Data collection

The following demographic and clinical data of patients with identified RGS8-Abs were collected from medical records: age, sex, clinical symptoms, disability at onset defined by the modified Rankin Scale, CSF analysis (white blood cell count and protein content), MRI findings, associated tumor when present, immunotherapy, and oncological treatment.

### Anti-RGS8 IgG isotyping and titration

The isotyping protocol is described in the Supplementary Methods and the titration of total IgG in serum and CSF was defined as the lowest dilution that allowed a positive signal on CBA, as previously described [5].

### Epitope mapping

Phage immunoprecipitation sequencing (PhIP-seq) [6] was used to epitope map RGS8-Abs using our custom library as previously described (https://github.com/derisilab-ucsf/PhIP-PND-2018) [7, 8]. Detailed protocol is provided in the Supplementary Methods.

### Pathological studies

For tumor analysis, 4-μm-thick formalin-fixed paraffin-embedded (FFPE) tissue sections were stained with hematoxylin phloxine saffron (HPS). A referent pathologist (A.T.G.) assessed the subtype of lymphoma according to the 2016 WHO classification using classical staining for HL diagnosis and, in situ hybridization to Epstein–Barr virus-encoded RNA (EBV ISH) [9].

Detailed chromogen IHC protocols and antibodies for HL diagnosis and subtyping are described in the Supplementary Methods. RGS8 expression was assessed using an automated IHC protocol using monoclonal commercial RGS8-Abs. Copy number alterations of the *RGS8* gene were assessed on 2 RGS8-nodular lymphocyte-predominant HL (NLPHL) samples using a RGS8 probe. A control cohort including two NLPHL cases and five cases of classical HL, distinct in the classification from the NLPHL type, was assessed.

### Standard protocol approval and patient consent

A written informed consent for sample analysis, medical research, and case publication was previously obtained from all patients. The study is part of the project Gene PNS (NCT03963700) and was approved by the Institutional Review Boards of the Hospices Civils de Lyon and UCSF (13–12,236).

## Results

### Identification of patients with RGS8-Abs

**Fig. 1:**
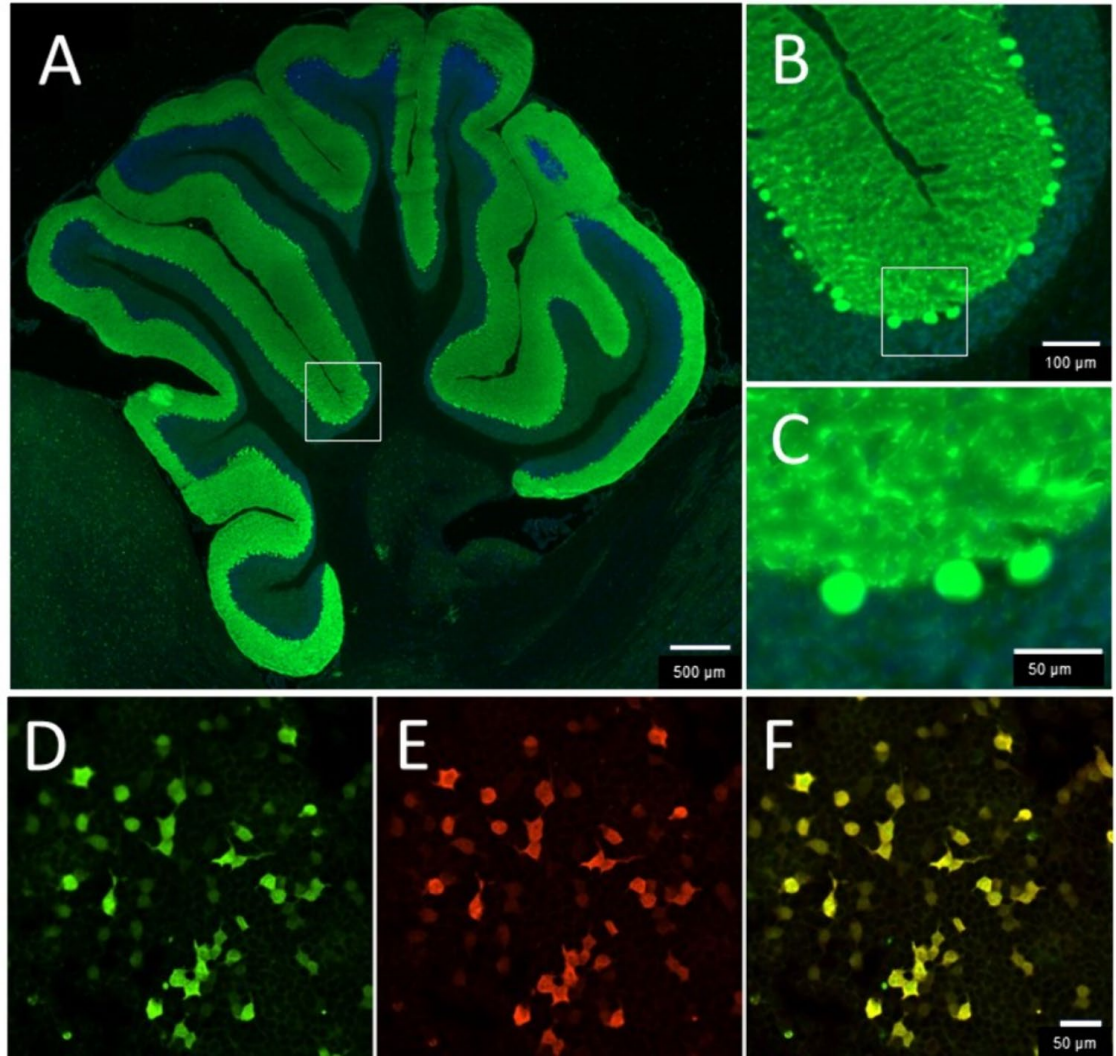
Detection of anti-RGS8 antibody in patient 1. **A**–**C**: Immunofluorescence on rat brain sections with a strong staining of the molecular layer of cerebellum and Purkinje cells. **A** Whole cerebellum, low magnification. Scale bar: 500 µm. **B** Zoom on the cerebellar cortex corresponding to the zone framed in white on A showing a selective staining of the molecular layer. Scale bar: 100 µm. **C** Strong immunofluorescence of Purkinje cells at high magnification. Zoom of zone framed in white on B. Sale bar: 10 µm. **D**–**F** Cell-based assay in HEK293 cells transfected to express RGS8 with pCMV-Entry-RGS8 plasmid and immunostained using a commercial antibody directed against Myc tag revealed in green (**D**). Staining with CSF of a patient with anti-RGS8 (**E**). Merged image (**F**) show co-localization of RGS8 (green) and patients’ antibodies (red)

Among the two biological collections, only three patients with RGS8-Abs in serum and/or CSF were identified using indirect IF (Fig. 1A–C) and CBA (Fig. 1D–F), showing positive concordant results. Two patients were identified in the biological collection of the French Reference Center on AE and PNS. The remaining patient, identified in the biological collection of UCSF, was already included in a previously published study [2]. As a comparison, during the same time span, 38 patients with Yo-PNS and 12 with DNER-ataxia were identified on the French cohort.

### Clinical description

#### Patient 1

A 50-year-old male with a previous history of type 1 diabetes presented with an 8-week progressive gait instability that impeded walking without assistance, subsequently followed by intense photophobia, double vision, and slurred speech. On examination, the patient exhibited dysarthria, horizontal nystagmus, diplopia without a clear limitation of extraocular movements, diffuse brisk reflexes without weakness, right-sided dysmetria, and severe gait ataxia. During his hospital stay, the CSF analysis showed < 5 nucleated cells/μL, mild total protein elevation (0.57 g/L), IgG intrathecal synthesis, and multiple oligoclonal bands exclusive to the CSF. Conversely, brain MRI and extensive laboratory tests including serum and CSF Abs against common onconeuronal antigens were unremarkable. A strong staining of the molecular layer of cerebellum and Purkinje cells was found using the indirect IF. Using the RGS8 CBA, RGS8-Abs were found in patient’s serum and CSF; the end-point dilution of the CSF was 1/2400, and that of the serum was 1/64,000, restricted to IgG1 subtype in both samples. Moreover, the line blot found a positive reactivity against RGS8. FDG-PET–CT revealed several right axillary hypermetabolic lymph nodes, and an excisional biopsy showed an NLPHL, stage I of Ann Arbor classification [10]. Of note, HLA haplotyping found the highly conserved 8.1 haplotype (A*01:01-B*08:01-DQB1*02:01-DRB1*03:01), complete patient’s haplotyping is reported in Supplementary Table 1. After excluding other causes of cerebellar dysfunction and upon suspicion of a paraneoplastic ACA, the patient received a 5-day course of intravenous immunoglobulins (IVIG), and a 3-day course of intravenous steroids. Due to the lack of clinical improvement, anti-CD20 therapy (rituximab) was administered, and 2 weeks later, he received a chemotherapy scheme comprising rituximab, cyclophosphamide, doxorubicin, vincristine, and prednisolone (R-CHOP). After presenting an initial mild improvement, the patient progressively worsened, and after 10 months of follow-up, despite a complete oncological remission, he remained severely disabled.

#### Patient 2

A 50-year-old male, with a previous history of Evans syndrome and who underwent splenectomy, experienced a 6-month sensation of shimmering lights, oscillopsia, and mild progressive gait instability. On examination, he had horizontal nystagmus, mild left arm dysmetria with kinetic intention tremor, and mild gait ataxia. The brain MRI was normal, but the CSF analysis showed an inflammatory CSF with unique oligoclonal bands. RGS8-Abs were found in the serum with an end-point dilution of 1/256,000 and were of IgG1, IgG2, and IgG4 subtypes. The line blot found a positive reactivity against RGS8. An FDG-PET–CT revealed hypermetabolic cervical, mediastinal, and mesenteric lymph nodes (Stage III of Ann Arbor classification) that revealed NLPHL upon biopsy. Complete HLA haplotyping is described in Supplementary Table 1. Of note, the patient harbored a class II HLA DRB1*07*01 allele. IVIG was administered upon suspicion of paraneoplastic ACA, as well as R-CHOP chemotherapy and intrathecal methotrexate for the NLPHL. The follow-up brain MRI, performed 11 months after diagnosis, showed already a moderate cerebellar atrophy. After 3 years of follow-up, the patient was in complete oncological remission and remained only mildly disabled, suggesting a good response to immunomodulating treatment.

#### Patient 3 (see also reference [8])

A 68-year-old male developed severe, rapidly progressive ataxia with oscillopsia and diplopia. The CSF analysis 4 months after symptom onset demonstrated a lymphocytic pleocytosis (white cell count of 104/µL, a high total protein elevation (0.85 g/L), multiple CSF-exclusive oligoclonal bands, benign cytology, and negative commercial onconeural autoantibody testing). RGS8-Abs were detected in CSF by PhIP-seq and confirmed by CBA. The brain MRI was normal. Whole-body FDG-PET–CT and scrotal ultrasound showed no evidence of malignancy. The patient was treated acutely with pulse glucocorticoids and IVIG with noticeable stabilization of the rapid worsening, followed by rituximab and 6 IV monthly pulses of cyclophosphamide, and then maintenance treatment with mycophenolate mofetil. A surveillance FDG-PET–CT performed after a 3-year follow-up showed mildly hypermetabolic lymph nodes above and below the diaphragm. An excisional biopsy performed on an axillary lymph node showed reactive lymphoid hyperplasia with benign histopathology and flow cytometry. Repeat FDG-PET–CT 1 year later revealed mildly hypermetabolic adenopathy in the pelvis. A second lymph node biopsy was inconclusive. At 5 years, he could ambulate short distances with a walker.

### RGS8-ACA is associated with NLPHL strongly expressing RGS8 protein

**Fig. 2:**
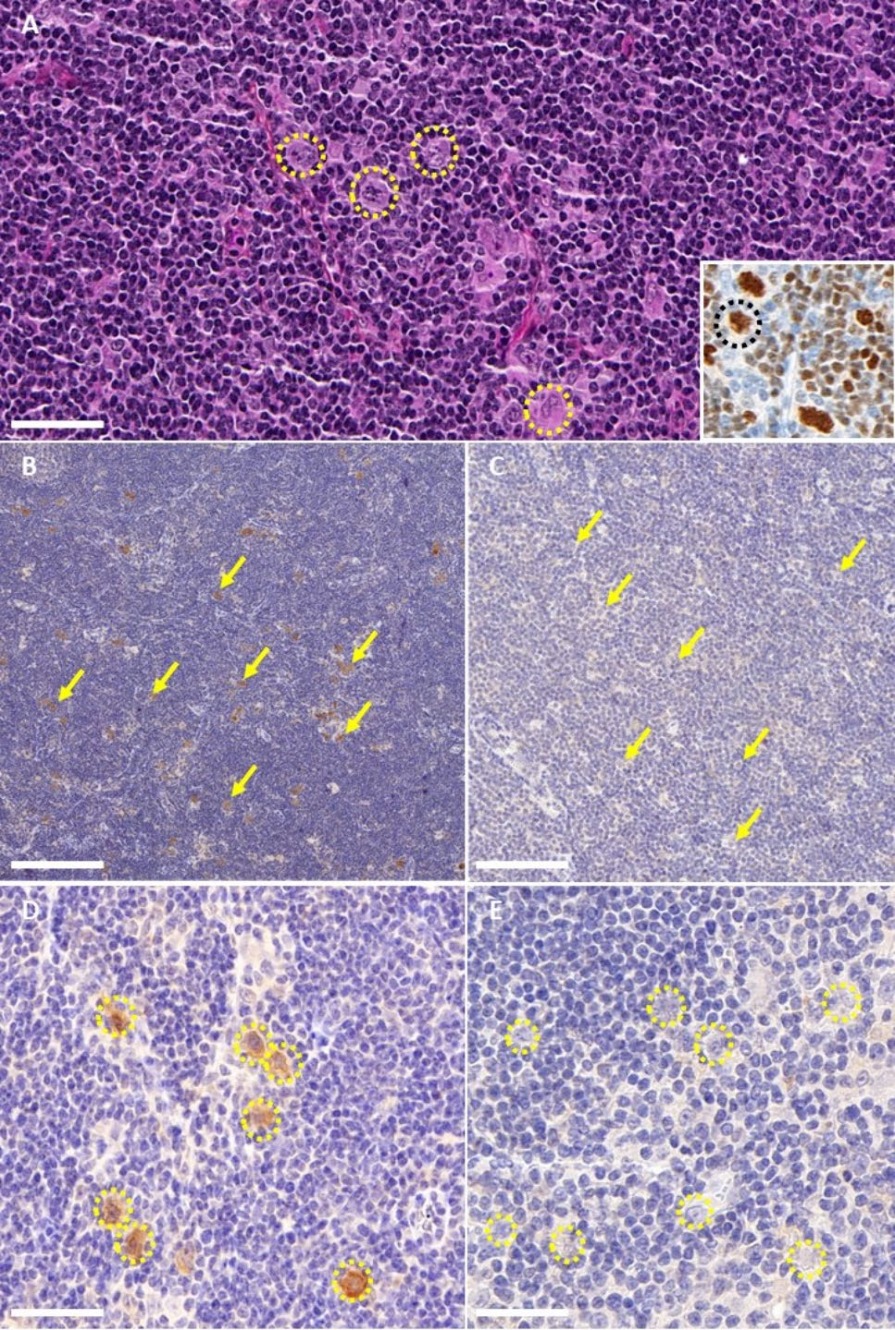
RGS8 expression in RGS8 patient’s nodular lymphocyte-predominant Hodgkin lymphoma (NLPHL) versus control NLPHL. **A** Typical histopathological presentation of NLPHL in high magnification. Yellow dotted line circles the hallmark cell of NLPHL, the so-called « popcorn cells». Scale bar: 50 µm. Insert on the bottom right corner shows Oct-2 staining at high magnification strongly marking the nuclei of popcorn cells, circled in black dotted line. Scale bar: 50 µm. **B** and **C** Comparative IHC staining with anti-RGS8 antibodies on RGS8 patient’s tumor on the left (**B**) and control tumor (**C**) on the right at low magnification, showing highly selective staining of the popcorn cells exclusively in RGS8 patient’s tumor. Yellow arrows point at some popcorn cells. Scale bar: 200 µm. **D** and **E** Comparative IHC staining with anti-RGS8 antibodies on RGS8 patient’s tumor on the left (**D**) and control tumor on the right (**E**) at high magnification, showing selective staining of the popcorn cells only in RGS8 patient’s tumor. Yellow dotted lines circle some « popcorn cells». Scale bar: 50 µm

Patient 1 and 2 presented with localized (patient 1) or extended (patient 2) NLPHL harboring the classical “popcorn cell” tumor cells (Fig. 2A), which is a histopathological hallmark of this rare subtype of HL. These cells were CD20, Oct-2 positive, MEF2B (Fig. 2A) but CD15 and CD30 negative, which ruled out classical HL. IHC staining revealed RGS8 cytoplasmic strong expression specifically in the “popcorn cell” of the tumor samples of both patients (Fig. 2B and D). No such expression was observed in the 2 NLPHL control tumors without associated paraneoplastic neurological syndrome nor in the 5 classical HL tested (Fig. 2C and E). Fluorescent in situ hybridization (FISH) was performed to assess the variation of copy number of *RGS8* but results were inconclusive because of the rarity of tumor cells (representing less than 1% of the cells) scattered in the inflammatory environment (data not shown).

### Anti-RGS8 autoantibodies recognize the same epitope in N-terminal region

**Fig. 3:**
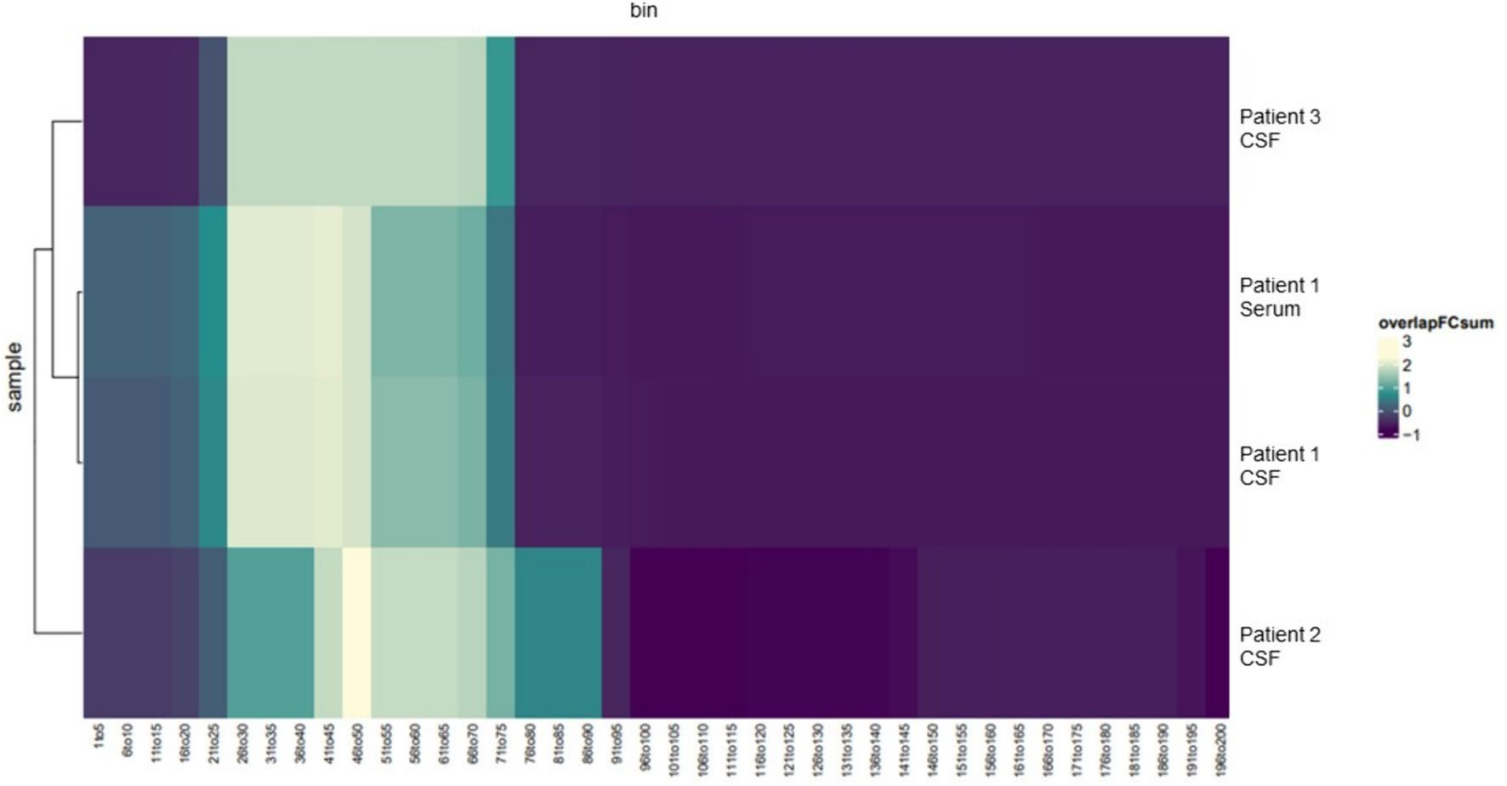
Epitope map of RGS8 peptides aligned to the full-length protein (Uniprot P57771; coverage is divided into five amino acid bins). CSF antibodies from all patients as well as patient’s 1 serum enriched an overlapping region toward the N-terminal domain

Epitope mapping using PhIP-Seq found that the CSF samples of the three patients targeted overlapping peptide regions close to the N-terminal part of the protein between amino acid 25 and 75. In patient 1, RGS8-Abs of both serum and CSF were assessed and enriched the same epitope (Fig. 3).

## Discussion

The present study described the clinical and tumoral features of three patients with ACA presenting RGS8-Abs. Currently, the number of known patients presenting a RGS8 syndrome is seven, but the associated tumors and clinical data have been described for only five patients[1, 2]; four were middle-age males and one was an older female [7]. All patients had a pure cerebellar ataxia with a severe course, except one who remained very mildly disabled (patient 2 of the present study). Interestingly, two patients in the present study, for whom the history was available, had at least one other prevalent autoimmune disease (type 1 diabetes for patient 1 and Evans syndrome for patient 2). Although incidence cannot be evaluated herein, we can postulate the extreme rarity of this syndrome, since only seven patients were identified retrospectively among the substantial biological collections of three countries (France, USA, and Germany [1, 2]).

Neuronal autoantibodies may predict the presence of underlying malignancies with variable accuracy. The recently updated diagnostic criteria for PNS stratified onconeural Abs into high-risk, intermediate-risk, and low-risk Abs according to the probability of associated neoplasm [11].

Among the seven identified patients with RGS8-Abs, the tumor identification was available for four, and all of them presented with a B-cell lymphoma; one with a diffuse large B-cell lymphoma of the stomach and three with an HL (one from the previous published study [1] and two herein). Hence, regarding the current diagnostic criteria [11], RGS8-Ab are at least Abs of intermediate risk of PNS, with a preferential association with B-cell lymphoma, particularly HL.

In the present study, the two RGS8-associated HL belonged to a particular subclass of HL called NLPHL, a rare subtype of HL accounting for ∼5% of all HL cases and which tends to have a markedly indolent course compared to other HL subtypes [12]. NLPHL has an overall similar incidence in all age groups, but, interestingly, all the patients with paraneoplastic anti-RGS8 ACA described so far are middle-aged or older patients. In the present series, patients presented with mildly locally extended diseases, as described in HL associated with DNER-Abs ACA, making the lymphoma diagnosis difficult [5]. Consequently, suspicion of RGS8-Abs ACA with lymphoma should be raised in cases of subacute cerebellar ataxia without evidence of an underlying malignancy, and patients should be evaluated accordingly.

Conversely to classical HL, NLPHL tumors poorly express CD15 and CD30, whereas B-cell markers (CD20, OCT2, MEF2B) and CD45a are expressed. The pathological hallmark of NLPHL is the presence of atypical tumor cells with polylobulated *nuclei* called “popcorn cells” surrounded by a rich environment of inflammatory cells [13]. The present study found a strong expression of the RGS8 antigen specifically in the scattered popcorn cells, but not in the microenvironment, only in patients with RGS8-Abs, which highlights a potential link between the lymphomagenesis and anti-RGS8 autoimmunity. As described in other cancer associated with PNS [14, 15], this strong expression of RGS8 only in the tumor cells of patients with PNS may be the trigger of the immune cross-reaction, and can thus be responsible for the death of Purkinje cells in which RGS8 is physiologically expressed. The strong expression of other onconeural antigen has been described to be linked to gene *locus* amplification in PNS [14, 15]. Unfortunately, we could not assess this question herein, since FISH was not feasible in lymphoma samples, as the specific isolation of popcorn cells among other lymphoma cells and reactive lymphocytes could not be successfully performed. Due to the same technical limitations, point mutation of the antigen in tumor cell, another mechanism of neoepitope generation, could not be studied.

The results of the first study that identified RGS8-Abs suggested the specificity of this autoantibody. Herein, RGS8-Abs enriched the same N-terminal region in the three patients tested by PhIP-Seq, suggesting a targeted immune response to a defined epitope, which provides additional information regarding the specificity of this autoantibody. Further studies are needed to definitively determine RGS8-Abs specificity and explore the pathobiological relevance of the target epitope. In this respect, it would be especially interesting to screen lymphoma patients without neurological symptoms for anti-RGS8 antibodies to determine the antibodies’ specificity. Such screening was unfortunately not feasible due to the rarity of this disease and the difficulty to collect samples prior to any chemotherapy in these patients.

In other PNS, the nature of the immunological reaction secondary to this immune tolerance breakdown is partially dictated by subcellular location of the antigen. Due to the intracellular location of the target [1], a potential pathogenic role of the antibody seems unlikely, whereas T-cell mediation of the cerebellitis is more probable. This latter hypothesis is supported by the poor response to immunotherapy of patient 1 and 3 even though it was administered early in the disease course. Similarly to other ACA with autoantibodies directed against intracellular targets, such as Yo-Abs [16] or Hu-Abs[17], the prognosis seems poor in the patients of the present series, irrespective of the therapeutic strategy. In all identified patients, the absence of reversibility of the symptoms suggests a prominent mechanism of neuronal death mediated by cytotoxic T cells. However, RGS8 is an important mediator of the mGluR1 pathway in Purkinje cells [18], and autoantibodies against mGluR1 have been described in patients with ACA with a high suspicion of a direct pathogenic role of the autoantibodies [19]; in this context, a direct role of RGS8-Abs remains to be carefully assessed in future studies. Besides its role in Purkinje cells, the putative role of RGS8 in lymphocytes remains to be studied as well as its potential implication in lymphomagenesis. Evidence in that respect remain scarce to date [20], but the quite specific association described herein along with the selective expression of the antigen in the most typical NLPHL cell strongly suggests a link between lymphomagenesis and RGS8 autoantigen that needs further exploration.

## Conclusion

The present results and those of the four cases previously described suggest that RGS8-Abs define a new PNS of extreme rarity found mostly in middle-aged male that associates pure cerebellar ataxia and lymphoma. RGS8-Abs are directed against an intracellular antigen specifically expressed in the tumor cells, the popcorn cells, NLPHL’s hallmark, and physiologically expressed in the Purkinje cells, which are targeted by the autoimmune reaction. As in other paraneoplastic ACA with intracellular antigen, RGS8-ataxia is probably T-cell mediated and antibodies are unlikely to be pathogenic. The disease course is consequently severe, and patients tend to exhibit a poor response to immune therapy.

## Supplementary Information

Below is the link to the electronic supplementary material.

Supplementary file1 (DOCX 21 kb)

## Data Availability

Anonymized data used for this study are available on request.
