# Supplementary material for: Anti-RGS8 paraneoplastic cerebellar ataxia is preferentially associated with a particular subtype of Hodgkin’s lymphoma
Source: J Neurol. 2024 Aug 29;271(10):6839–46. doi: 10.1007/s00415-024-12618-4 (PMC11447075; doi:10.1007/s00415-024-12618-4)
Supplement: Supplementary file 1 — Supplementary file1 (DOCX 21 kb) [file 415_2024_12618_MOESM1_ESM.docx]

**ANTI-RGS8 PARANEOPLASTIC CEREBELLAR ATAXIA IS ASSOCIATED WITH A PARTICULAR SUBTYPE OF HODGKIN’S LYMPHOMA**

Supplementary material

**Supplementary method**

Anti-RGS8 titration

Either 100 or 200 μL (according to whether 96 or 24-well plates were used) of the IgG solution of interest (diluted 1/1,000 for IgG1 and IgG3, and 1/250 for IgG2 and 1/500 for IgG4) was incubated with RGS8-transfected HEK293 cell for 1 hour at room temperature in the dark. A secondary goat anti-mouse antibody (diluted 1/1,000) coupled to a fluoro- chrome was added and then incubated for 1 hour at room temperature in the dark.

Antibodies and probes references

| Isotpyping | |
| --- | --- |
| IgG1 | mouse anti-human IgG1 CH2 domain; Bio-Rad, Hercules, CA, USA |
| IgG2 | purified mouse anti-human IgG2 clone G18-21 [RUO]; BD Biosciences, San Jose, CA, USA |
| IgG3 | mouse anti-human IgG3; Bio-RAD |
| IgG4 | purified mouse anti-human IgG4 clone G17-4 [RUO]; BD Biosciences |
| Secondary antibody | Alexa Fluor 555; ThermoFisher Scientific |
| IHC | |
| CD3 | A0452, DAKO, Agilent, Santa Clara, CA, USA) |
| CD20 | MO755, DAKO |
| CD23 | SP23, Invitrogen |
| Ki-67 | 790-4286, Ventana, Oro Valley, AZ, USA |
| CD30 | MO755, DAKO |
| CD15 | 760-2504, Ventana |
| PAX5 | 790-4420, Ventana |
| Oct-2 | 760-4447, Ventana |
| MEF2B | HPA004734, Sigma, Saint-Louis, MO, USA |
| MUM1 | MUM1p, DAKO |
| IgD | 760-4444, Ventana |
| BOB1 | 760-4593, Ventana |
| EMA | 760-4463, Ventana |
| PD1 | 760-4895, Ventana |
| EBV ISH | 800-2842, Ventana cell marker |
| CBA | |
| Anti-Myc | C3956, Sigma |
| Secondary antibody | A21433, Fisher Scientific, Hampton, NH, USA |
| Secondary antibody | A11034, Fisher Scientific, Hampton, NH, USA |
| OCS | |
| commercial rabbit antibody targeting RGS8 | PA5-85008, Invitrogen |
| mouse anti-Calbindin D-28K antibody | 214011, Synaptic system, Göttingen, Germany |
| Alexa-Fluor 555 goat anti-rabbit IgG | 10143952 Fisher scientific, Illkirch, France |
| Alexa-Fluor 647 goat anti-mouse IgG2a | A-21241, Invitrogen |
| Alexa-Fluor 488 goat anti-human IgG | A11013, Invitrogen, Courtaboeuf, France |
| Western blot | |
| anti-Calpain antibody | C5986, Sigma-Aldrich |
| mouse anti-PanCadherin antibody | C1821, Sigma-Aldrich |
| HRP-coupled goat anti-rabbit IgG | 111-036-003, Jackson ImmunoResearch, Ely, UK |
| HRP-coupled goat anti-mouse IgG | 115-036-003; Jackson ImmunoResearch |
| FISH probe | |
| RGS8 FISH probe | RGS8-20-OR, Empire Genomics, Buffalo, NY, USA) |

Phage Immuno-Precipitation

Briefly, the CSF of patients was incubated with the 10^10^ plaque forming units of the phage library, antibodies were enriched with protein A/G magnetic beads, and antibody-bound phage was amplified in *Escherichia coli* before a second round of immunoprecipitation. Enriched phage lysates were adaptor ligated and barcoded prior to pair-end sequencing on an Illumina Novaseq (Illumina, San Diego, CA, USA). Reads were trimmed, aligned at the amino-acid level using RAPSearch, and normalized to sequencing depth to generate reads per 100,000 bases (RPK) for each sample. Enriched peptides were identified by calculating the fold-change of normalized counts between samples immunoprecipitated with CSF or magnetic beads only. Enriched peptides were aligned to RGS8 (UniprotKB P57771), and enrichment was calculated in 5-amino-acid bins for each patient sample.

Supplementary table

**Supplementary Table 1. Patient 1 and 2 complete HLA haplotyping.**

|  | Patient 1 | | Patient 2 | |
| --- | --- | --- | --- | --- |
|  | Allele 1 | Allele 2 | Allele 1 | Allele 2 |
| Locus A | A*01:01 | A*02:01 | A*24:02 | A*29:02 |
| Locus B | B*08:01 | B*51:01 | B*39:06 | B*44:03 |
| Locus C | C*07:01 | C*16:02 | C*07:02 | C*16:01 |
| Locus DRB1 | DRB1*03:01 | DRB1*11:03 | DRB1*07:01 | DRB1*08:01 |
| Locus DRB3 | DRB3*01:01P | DRB3*02:02P |  |  |
| Locus DRB4 |  |  | DRB4*01 |  |
| DQB1 gene | DQB1*02 | DQB1*03 | DQB1*02 | DQB1*04 |
| DQA1 gene | DQA1*05:01 | DQA1*05:05 | DQA1*02 | DQA1*04 |
| DPB1 gene | DPB1*04 | DPB1*09 | DPB1*01:01 |  |
